# Identification of an env-defective HIV-1 mutant capable of spontaneous reversion to a wild-type phenotype in certain T-cell lines

**DOI:** 10.1186/1743-422X-11-177

**Published:** 2014-10-06

**Authors:** Yudong Quan, Hongtao Xu, Victor G Kramer, Yingshan Han, Richard D Sloan, Mark A Wainberg

**Affiliations:** Lady Davis Institute for Medical Research, Jewish General Hospital, McGill University AIDS Centre, 3755 Cote Sainte Catherine, Montreal, QC H3T 1E2 Canada; Division of Experimental Medicine, Faculty of Medicine, McGill University, Montréal, Québec Canada; Department of Microbiology and Immunology, Faculty of Medicine, McGill University, Montréal, Québec Canada

**Keywords:** Defective virus, Reversion, HIV, Cell-associated transmission

## Abstract

**Background:**

Attempts to eradicate HIV from cellular reservoirs are vital but depend on a clear understanding of how viral variants are transmitted and survive in the different cell types that constitute such reservoirs. Mutations in the env gene of HIV may be able to exert a differential influence on viral transmission ability in regard to cell-free and cell-associated viral forms.

**Methods:**

The ability of HIV containing an env G367R mutation in cell-free and cell-associated viruses to cause infection and to revert to wild-type was measured using several T cell lines. To determine factors that might potentially influence the reversion of G367R, we studied each of entry inhibitors, inhibitors of cellular endocytosis, and modulators of cell growth and activation.

**Results:**

We demonstrate that an HIV-1 variant containing a G367R substitution within the CD4 binding site of gp120 was non-infectious as free virus in culture but was infectious when infected cells were co-cultured with certain T cell lines or when cells were transfected by a relevant proviral plasmid. Differences in viral infectivity by cell-associated G367R viruses were determined by the type of target cell employed, regardless which type of donor cell was used. Reversion was slowed or inhibited by entry inhibitors and by inhibitors of cellular endocytosis. Interleukin 2 was able to block G367R reversion in only one of the T cell lines studied but not in the other, while phorbol 12-myristate 13-acetate (PMA) inhibited G367R reversion in all the T cell lines.

**Conclusions:**

Env-defective HIV may have a different phenotype as cell-free versus cell-associated virus. The persistence of defective forms can potentially lead to the emergence of virulent forms. The heterogeneity of cell types that constitute the HIV reservoir can contribute to viral variability, even among similar types of cells. This is the first demonstration of a mutation in the HIV envelope, i.e. G367R, that can compromise infection by cell-free virus but less severely by cell-associated virus and that does so in a cell type-dependent manner.

## Introduction

HIV-1 can be efficiently transmitted as free virus or directly between cells via cell-cell contact, each of which involves receptor and coreceptor binding. Although cell-free HIV may be used to initiate new infections in tissue culture, cell-to-cell transmission is considered to be more physiologically relevant and efficient [1–4].

HIV-1 entry into target cells is believed to be a multistep process initiated by binding between the envelope protein gp120 and cell surface CD4. This binding then triggers conformational changes of gp120 that lead to a second-step interaction between gp120 and a coreceptor such as CXCR4 or CCR5 [5–7], resulting in viral membrane fusion with the cellular plasma membrane [8]. In addition to viral proteins, several host proteins including the histocompatibility complex can influence HIV infectivity [9, 10]. However, it has also been reported that HIV can enter target cells via a CD4-independent or coreceptor-independent mechanism [11–13], potentially broadening the spectrum of cells that HIV is able to infect. Thus, the process of HIV entry is complex and can involve different channels.

Meanwhile, the fitness of HIV is critical for transmission and pathogenesis. Unlike many viruses, HIV has very high genetic variability and evolves quickly. The viral population in an infected individual is highly heterogeneous. Therefore, HIV-1 infected individuals may contain diverse viral swarms termed quasispecies that are similar but genetically distinct [14, 15]. Large numbers of mutations, including those responsible for drug resistance, may exist in the viral population of infected individuals [16]. A major proportion of human immunodeficiency virus among quasispecies may be defective due to the spontaneous generation of lethal mutations. However, defective proviral mutants may still be able to play a role in HIV pathogenesis, e.g. through recombination and rescue of drug resistance phenotypes [17] and viral recombination may take place with defective viral forms among the quasispecies and increase viral fitness as well as transmission. There are reports that a highly infectious virus-producing cell line may contain five copies of the HIV genome, none of which is infectious individually [18]. Increased efficiency of HIV transmission may increase the likelihood that target cells become infected by multiple virions and increase the chances of viral recombination [19–22]. This, in turn, could facilitate viral escape from selection pressure by drugs and the immune system [16, 23].

In regard to transmission, the viral envelope protein is not only responsible for viral entry but also modulates certain functions of host cells that facilitate infection. HIV pseudotyped with VSV-G cannot successfully infect resting T cells [24] and mutations in the viral envelope proteins may affect viral infectivity through different mechanisms. Certain mutations, including those at positions G367R and D368R in the CD4 binding site (CD4bs) of gp120, may cause the virus to become non-infectious [17, 25–27].

Most HIV research has involved the use of cell-free viruses, although it is known that it is easier to isolate HIV from cocultures of infected lymphocytes than plasma. Although the reasons for this are unclear, it is possible that HIV variants that are harbored within cells can be transmitted more efficiently than cell-free forms at least until faster-growing viruses ultimately emerge.

We have previously documented that a substitution in Env at position G367R can result in viral non-infectiousness and that this deficit can be rescued by recombination. One of the purposes of the current study was to further characterize this deficit and provide new information on its physiological importance. In this manuscript, we show that the defectiveness of the G367R mutation is more severe in the context of free virus than cell-associated virus, but only in certain types of cells. We have studied the reversion of env-defective mutants in several T cell lines and obtained different results with cell-free vs cell-associated viruses in regard to env defectiveness. Here, we report that some env mutants may perform differently in cell-free versus cell-to-cell transmission and that a G367R mutant can spontaneously revert in some T cell lines. Viruses containing this mutation, that is located within the CD4bs of gp120, are non-infectious in culture when cell-free viruses are employed as reported previously [17]. We also report that G367R reversion was slowed or inhibited by HIV entry inhibitors, such as the gp120 binding agent DS003 and the CXCR4 antagonist AMD3100, as well as by inhibitors of endocytosis at sub-toxic concentrations. Interestingly, interleukin 2 (IL2) can block G367R reversion in MT2 cells but not in SupT1 cells, while PMA is able to inhibit reversion in both cell types, suggesting that complex mechanisms are involved in the reversion process.

## Materials and methods

### Cells

MT2, MT4, Jurkat, CEM, PM1 and SupT1 cells were all obtained through the NIH AIDS Research and Reference Reagent Program. All these cell types can efficiently support CXCR4-dependent HIV replication, and both MT2 and MT4 cells are standards for HIV replication studies. All cells were maintained in RPMI 1640 medium (Invitrogen) supplemented with 10% fetal bovine serum (Invitrogen), 1% L-glutamine, and 100 units/ml penicillin/100 μg/ml streptomycin under 5% CO_2_ at 37°C. 293 T and TZM-bl cells were maintained in Dulbecco modified Eagle medium (Invitrogen). Culture media were changed every 3 days.

### Viruses

The HIV-1 HxB 2D clone was used as wild type (WT) virus. A defective env G367R mutant virus was produced by transfecting a proviral plasmid, that contained a mutation at position G367R in the CD4 binding site of gp 120, and that has been generated by PCR based site directed mutagenesis [17]. pVPack-VSV-G (Stratagene), which encodes the vesicular stomatitis virus (VSV) envelope glycoprotein, was used at 1:1 to produce VSV-G pseudotyped viruses. G367R mutant viruses and VSV-G pseudotyped viruses were generated in 293 T cells by transfection from the proviral plasmids with lipofectamine 2000 as recommended by the manufacturer (Invitrogen). At 48 hours after transfection, viruses were harvested, analyzed for p24 antigen and stored at −80°C.

R5 tropic subtype C Indie viruses were generated in 293 T cells by transfection from the Indie C proviral plasmid [28], also using lipofectamine 2000 as recommended by the manufacturer.

Virus stocks of WT were amplified in MT2 cells or in cord blood mononuclear cells (CBMCs), obtained through our Hospital Department of Obstetrics. Virus stocks were kept at −70°C until use.

### Viral infection

2-5×10^5^ cells were infected with 0.5 ml of culture fluid containing ~500 ng of p24 of HIV for 3 hr at 37°C. Cells were washed three times with RPMI 1640 medium immediately following incubation to remove unbound virus and were resuspended in RPMI 1640 medium and split into wells at appropriate concentrations. Cytopathic effects (CPE) were monitored by microscopy and supernatants were evaluated for p24 antigen at 3 day intervals (Biomerieux, Netherlands).

For infection of 293 T or TZM-bl cells, 10^5^ cells plated the previous day were infected overnight by 0.5 ml of VSV-G pseudotyped viruses. Supernatants were removed and washed once and fresh medium was added. Supernatants were collected and cells were split at 3 day intervals and monitored for p24 antigen.

### Viral infection by coculture

10^4^ 293 T or TZM-bl cells infected with pseudotyped G367R virus in a donor coculture were mixed with 5×10^4^ target cells. Alternatively, 10^5^ MT4 cells infected with pseudotyped G367R virus in a donor coculture were mixed with 2-4×10^5^ MT2 cells. CPE and levels of p24 antigen in supernatants were monitored to verify efficiency of infection and reversion.

### HIV inhibitors and endocytosis inhibitors

The HIV entry inhibitors AMD3100, maraviroc, and DS003 were all obtained through the NIH AIDS Research and Reference Reagent Program. The endocytosis inhibitors chlorpromazine hydrochloride, Genistein and methyl-β-cyclodextrin were purchased from Sigma-Aldrich, Inc. Phorbol 12-myristate 13-acetate (PMA) was purchased from Cayman Chemical, Ann Arbor, MI.

## Results

### The env mutant G367R is defective in cell-free infection but can replicate and revert to wild type spontaneously in the MT2 infected T cell line

**Figure 1:**
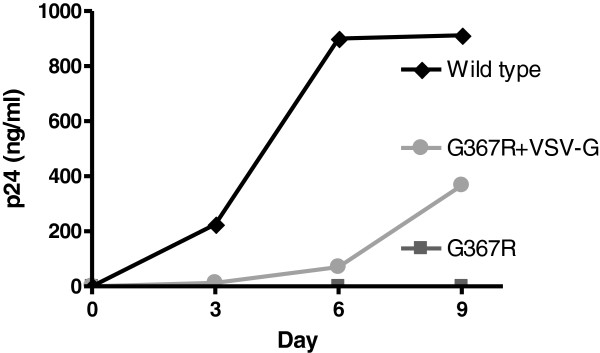
**p24 production in MT2 cells after infection by G367R virus pseudotyped with VSV-G, G367R or wild type viruses.** 3×10^5^ MT2 cells were infected for 3 hours at 37°C, then washed with medium, and split into 3 wells for growth.

We previously reported that viruses containing the env mutant G367R are defective in cell-free infection [17], similar to what has been observed for a mutation at position 368, D368R [25–27]. Theoretically, expected reversion rates should be about 10^−5^ at a given site in single round infection studies, if the error rate of HIV reverse transcriptase is estimated at around 5×10^−4^. Thus, reversions might only be expected at a rate of 5×10^−4^, i.e., it would only be observed in a proportion of experimental studies when 10^5^ cells or fewer are seeded after infection at appropriate viral concentrations. However, we found higher reversion rates than expected when we employed the defective G367R virus that was pseudotyped with VSV-G and then used to infect MT2 cells (Figure 1 and Table 1). Figure 1 shows that the increase in p24 in the G367R-infected samples was slower than occurred when WT virus was used to mediate infection. This is not unexpected, considering that only a portion of cells are infected in a single round infection because of defective progeny viruses. However, CPE appeared in all the samples within 1–3 weeks, contrary to expectations. Reversion was confirmed by the fact that the progeny viruses gained the ability to participate in cell-free infections during second round infection experiments. This was demonstrated as well by documentation of a wild-type genotype, as shown by sequencing of the viral env gene (see below). In contrast, G367R alone (i.e., not pseudotyped) was noninfectious (Figure 1), consistent with our previous report [17]. Similar results were obtained for all samples tested over repeat experiments that involved seeding either 5×10^4^ or 10^4^ infected cells (Table 1). Thus, the reversion rate of G367R seems higher than the RT error rate over a single round of infection. As a consequence, we next investigated this phenomenon using a number of different cell types.

**Table 1:** **G367R + VSV-G reversion in different cell types**

Cells and numbers		Viruses used and time of appearance of CPE			
		G367R		G367R + VSV-G	
		CPE^a^	p24	CPE	p24
MT2	10^5^	No^b^	0	8-12d (3/3)^c^	>600 ng/ml^d^
	5×10^4^			9-15d (6/6)	>600 ng/ml
	10^4^			11-21d (24/24)	>600 ng/ml
	10^2^			21d (16/16)	>600 ng/ml
MT4	10^5^	No	0	14d (2/3)	>600 ng/ml
SupT1	10^4^	No	0	21d (6/6)	>600 ng/ml
	5×10^2^			35d (24/24)	>600 ng/ml
PM1	10^4^	No	0	(0/6)	0

Cells were incubated with viruses at 4-fold dilution for 3 hours, washed and resuspended in fresh media, and split into wells at appropriate concentrations.

^a^CPE = cytopathic effect.

^b^No = CPE not observed over 28 days.

^c^number (d = day) represents the time of first appearance of CPE in the last sample analyzed over 28 days. Numbers (in parentheses) designate numbers of wells in which CPE was observed.

^d^evaluated during 28 days after infection. p24 values of CPE negative samples were lower than listed.

### The G367R reversion is both cell-type dependent and viral infection dose-dependent

**Figure 2:**
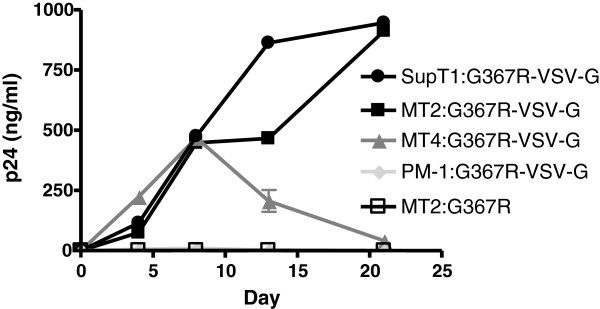
**p24 production in MT2, MT4, SupT1 and PM-1 cells after infection by G367R virus pseudotyped with VSV-G.** Cells were infected for 3 hours at 37°C, then washed with medium, and 10^5^ cells were split into wells in triplicate for growth assays.

Four T cell lines, MT2, MT4, SupT1 and PM-1, were used to investigate the mechanism(s) whereby G367R can achieve reversion. All of these cells express both the CD4 receptor and the CXCR4 coreceptor, while PM-1 cells also express the CCR5 coreceptor. Variable amounts of virus and cell numbers were used in order to estimate the reversion rate. The growth curves and extent of CPE of G367R mutated virus in each of MT2, MT4, SupT1 and PM-1 cells are displayed in Figure 2 and in Table 1. The results show that reversion of G367R was cell-type dependent, with reversion occurring very frequently in both MT2 and SupT1 cells but rarely in MT4 and PM-1 cells (Tables 1 and 2). The initial infection of MT4 by pseudotyped G367R was successful on the basis of p24 value but viral replication then decreased gradually while PM-1 cells were not as sensitive to infection as MT4 or MT2 cells (Figure 2). Sequencing of the env gene in all the reverted MT2 and SupT1 cells, regardless the number of infected cells that were seeded demonstrating that G367R had reverted from GGA AGG GAC CCA to GGA GGG GAC CCA, corresponding to a change in amino acid sequence from GRDP to GGDP within the CD4 binding site (Table 3).

**Table 2:** **Dose dependent reversion of G367R + VSV-G in MT2 and MT4**

Cells and numbers		Viruses used and time of appearance of CPE				
		Virus dilution^a^	G367R		G367R + VSV-G	
			CPE^b^	p24	CPE	p24
MT2	10^4^	4×	(0/24)^c^	0	14d (24/24)	>600 ng/ml^d^
	500	4×			14d (16/16)	>600 ng/ml
	500	12×			21d (14/16)	>600 ng/ml
	100	36×			28d (15/16)	>600 ng/ml
MT4	10^5^	4×	(0/3)	0	14d (2/3)	>600 ng/ml
	5000	12×			(0/3)	<50 ng/ml
	5000	36×			(0/3)	<50 ng/ml

MT2 or MT4 cells were incubated with viruses at different dilutions for 3 hours, washed and resuspended in fresh media, and split into wells at appropriate concentrations.

^a^virus dilution used for infection.

^b^CPE = cytopathic effect.

^c^number (d = day) represents the time of first appearance of CPE in the last sample analyzed over 28 days. Numbers (in parentheses) designate the numbers of samples in which CPE was observed.

^d^evaluated during 28 days after infection. p24 values of CPE negative samples were lower than listed.

**Table 3:** **Sequences of reverted G367R env around CD4 binding site**

	HIV Env	365	366	367	368	369	370	
HXB2D	A AGA GAA CAA TTT GGA AAT AAT AAA ACA ATA ATC TTT AAG CAA TCC	TCA	GGA	GGG	GAC	CCA	GAA	ATT GTA CGC
G367R	A AGA GAA CAA TTT GGA AAT AAT AAA ACA ATA ATC TTT AAG CAA TCC	TCA	GGA	**A**GG	GAC	CCA	GAA	ATT GTA CGC
R1-MT2	A AGA GAA CAA TTT GGA AAT AAT AAA ACA ATA ATC TTT AAG CAA TCC	TCA	GGA	GGG	GAC	CCA	GAA	ATT GTA CGC
R2-MT2	A AGA GAA CAA TTT GGA AAT AAT AAA ACA ATA ATC TTT AAG CAA TCC	TCA	GGA	GGG	GAC	CCA	GAA	ATT GTA CGC
R3-MT2	A AGA GAA CAA TTT GGA AAT AAT AAA ACA ATA ATC TTT AAG CAA TCC	TCA	GGA	GGG	GAC	CCA	GAA	ATT GTA CGC
R1-SupT1	A AGA GAA CAA TTT GGA AAT AAT AAA ACA ATA ATC TTT AAG CAA TCC	TCA	GGA	GGG	GAC	CCA	GAA	ATT GTA CGC
R2-SupT1	A AGA GAA CAA TTT GGA AAT AAT AAA ACA ATA ATC TTT AAG CAA TCC	TCA	GGA	GGG	GAC	CCA	GAA	ATT GTA CGC

HXB2D represents wt, G367R represents the mutant plasmid, R1-3 represent the reverted viruses recovered, respectively, from G367R + VSV-G infected MT2 or SupT1 cells when seeded at cell numbers of 10^5^, 10^4^ or 10^2^.

The time of reversion was closely related to the numbers of cells seeded after 3 hours of infection and the seeding of higher numbers of cells in each well led to faster reversion. Reversion of HIV from the same batch of infected cells occurred within 2 weeks when 10^4^ infected cells were seeded but only after 3 weeks if 100 infected cells were seeded (Table 1). Therefore, the reversion rate in one round, based on an inoculum of 100 cells was at least 500-fold higher than that expected only on the basis of the RT mutation rate and the estimation that each cell is successfully infected by a single virus. Otherwise, a single cell would need to be infected by 500 viruses simultaneously, representing an impossible situation.

Virus concentration may also affect the rate and time of reversion as shown in Table 2. Reversion appeared faster in samples infected by higher rather than lower amounts of virus. There was no reversion in MT4 cells when diluted pseudotyped G367R was used to initiate infection. This is reasonable because the fewer the viruses, the fewer infected cells in the first round. These findings indicate that reversion of the mutant G367R virus is unlikely to be generated over a single round of infection and that multiple rounds of viral replication may be needed in order to increase the chances for a revertant to emerge.

Similar results to those obtained with PM-1 cells were obtained with both Jurkat cells, CEM cells and CBMCs when infected by the G367R mutant pseudotyped with VSV-G (data not shown).

### The G367R reversion is facilitated by viral transmission between cells

To further investigate the mechanisms involved, we carried out several experiments to eliminate interference, if any, by the VSV-G protein and by HTLV-1 that is constitutively produced by MT2 cells. First, MT2 cells were directly transfected with the G367R proviral plasmid instead of by infection. Reversion in the G367R transfected MT2 cells was comparable in terms of time required to that of infection by the VSV-G pseudotyped G367R mutant, indicating that reversion does not seem to be influenced by the VSV-G protein. The fact that SupT1 cells infected by G367R pseudotyped with VSV-G reverted in the same manner as seen with infection of MT2 cells negates the likelihood that the HTLV-1 in the MT2 cells contributed to the reversion. In support of this, MT2 cells that were infected by R5 tropic subtype C Indie viruses that had been pseudotyped with VSV-G did not produce progeny that could infect MT2, MT4 or SupT1 cells, because such progeny viruses are unable to infect these cells in second round infections (data not shown). Therefore, these results do not support the idea that the HTLV-1 envelope protein can pseudotype with HIV, since, HTLV-1 pseudotyped R5 viruses should also be infectious were this to occur, consistent with our recent report [29].

Finally, supporting evidence comes from results obtained with 293 T and TZM-bl cells that were infected by VSV-G pseudotyped G367R virus and then cocultured with MT2 or MT4 cells. The results all indicate that reversion requires viral transmission through a cell-to-cell route, as shown in Table 4. MT2 cells cocultured with the G367R positive 293 T or TZM-bl cells developed CPE within about 1–2 weeks while the cells infected by G367R positive supernatants did not. Reversion in the cocultured MT2 cells was confirmed over a second round of cell-free infection. In contrast, MT4 cells that were cocultured with G367R positive 293 T or TZM-bl cells did not develop CPE over a 4 week period. However, phenotypic reversion did occur in the G367R positive TZM-bl cells that were maintained for up to 10 weeks in culture, since both supernatants and cocultures were infectious at the time points that were tested (Table 4). In contrast, the infection of 293 T cells did not lead to reversion on the basis of the infectivity of supernatants and p24 values that decreased at week 10 (data not shown). This indicates that G367R mutant virus can infect or reinfect TZM-bl cells but cannot infect 293 T cells.

**Table 4:** **G367R infectivity in cell cocultures**

Weeks	G367R + VSV-G		CPE^a^	
			MT2^b^: 10^5^	MT4: 10^5^
2^c^	293 T	SN^d^	-^e^	-
		Co	+	-
	TZM-bl	SN	-	-
		Co	+	-
4	293 T	SN	-	-
		Co	+	-
	TZM-bl	SN	-	-
		Co	+	-
6	293 T	SN	-	-
		Co	+	-
	TZM-bl	SN	-	-
		Co	+	-
10	293 T	SN	-	-
		Co	+	-
	TZM-bl	SN	+	+
		Co	+	+

293 T or TZM-bl donor cells were infected by pseudotyped G367R viruses. The infected cells or supernatants were used to infect target cells at different time points.

^a^CPE = cytopathic effect.

^b^target cell type and numbers used for infection.

^c^number of weeks after infection of donor cells.

^d^SN = supernatants of infected donor cells were used for infection, Co = coculturing target cells with infected cells at a ratio of 1:5.

^e^−designates that CPE was not observed over 14 days. + designates that CPE was observed within 14 days.

### The spontaneous reversion of G367R mutant involves cell-to-cell transmission and the efficiency of reversion is determined by the type of target cell employed

**Figure 3:**
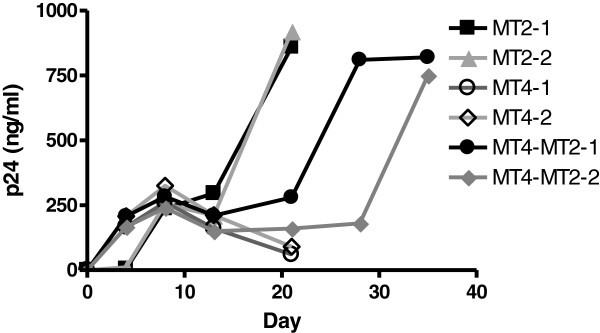
**p24 production in MT2-MT4 cell cocultures after infection by G367R pseudotyped with VSV-G viruses.** MT2 or MT4 cells were infected for 3 hours at 37°C, then washed with medium, and 10^5^ cells were split into 2 wells for growth assays. 10^5^ infected MT4 cells after 7 days from each well were split into 2 wells and mixed with fresh 2×10^5^ MT2 cells. Two curves are shown separately because the values of the duplicate cocultures were different.

Next, we investigated whether donor or target cells determine the efficiency of reversion of the G367R mutant. As shown above, coculturing G367R positive 293 T and TZM-bl cells, but not their supernatants, with MT2 or MT4 cells confirmed that the cell-associated the G367R virus can infect MT2 cells and revert to WT but cannot infect MT4 cells (Table 4). This result indicates that the type of target cell is important in determining the reversion of G367R mutant. The fact that MT4 cells infected by diluted G367R viruses that were pseudotyped with VSV-G did not revert is also in agreement with this result (Table 2). This finding also indicates that the mutant G367R HIV-1 cannot transmit if MT4 cells serve as both donor and target cells, as p24 values decreased gradually (Figure 3). In contrast, MT4 donor cells can transmit HIV-1 G367R mutant to MT2 target cells. In our experiments, CPE appeared after about 3 weeks in cocultures of MT4 cells containing G367R provirus, i.e., MT4 donor cells with uninfected MT2 cells. Reversion of phenotype was confirmed by a steep increase in p24 values in culture fluids (Figure 3) and the ability of the supernatants to participate in a second round of infection via cell-free transmission (data not shown). Thus, the G367R mutant has no deficit in ability to bud from MT4 cells. Moreover, it can be concluded that the efficiency of the G367R reversion is completely determined by the type of target cell used and not by donor cells.

### Entry inhibitors can delay G367R reversion

**Figure 4:**
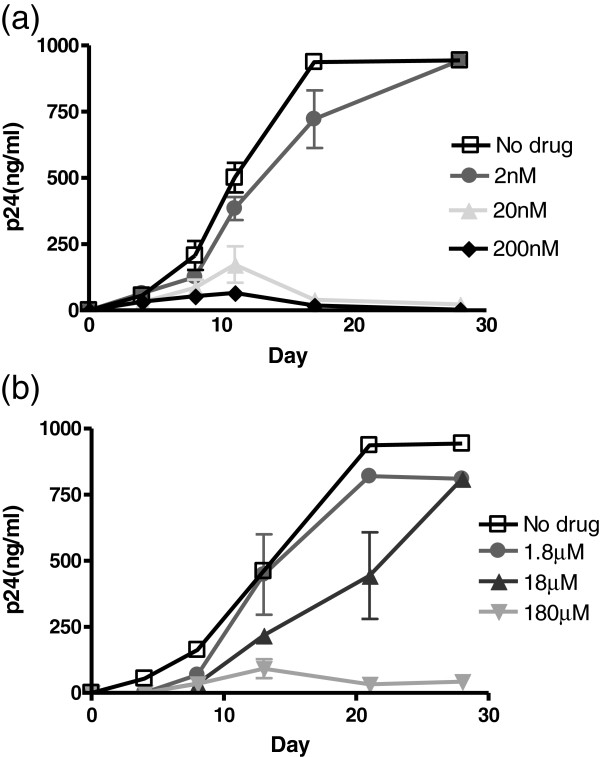
**p24 production in MT2 cells after infection by G367R virus pseudotyped with VSV-G viruses in the presence of entry inhibitors.** MT2 cells were infected for 3 hours at 37°C, then washed with medium, and 10^5^ cells were split into wells in triplicate for growth in the presence or absence of DS003 **(a)** or AMD3100 **(b)**.

To determine whether the observed reversion was related to virus entry, three entry inhibitors were employed to determine their effect on transmission and reversion. A gp120 binding inhibitor DS003 and a CXCR4 inhibitor AMD3100 were used in MT2 cultures infected by G367R viruses pseudotyped with VSV-G. A CCR5 inhibitor Maraviroc was used as a control. The results showed that DS003 was not able to inhibit G367R reversion when used at a fairly low concentration, i.e. 2 nM, a concentration around the IC_50_ (3.1 nM as determined in an assay of cell-free infection with WT virus) (Figure 4a). However, DS003 was able to inhibit G367R reversion at concentrations of 20 nM and 200 nM, since the p24 values in culture supernatants increased by day 14 and then decreased. In contrast, p24 values of all the samples tested after withdrawal of DS003 at day 7, continued to increase (data not shown) and CPE developed within 3 weeks. However, these viruses developed resistance quickly since CPE appeared in one of three experiments at each of 20 nM and 200 nM DS003 at 5 weeks after infection, accompanied by an increase in p24 value (Table 5). The resultant progeny was resistant to DS003 in a second round of cell-free infection (data not shown).

**Table 5:** **Inhibition of G367R virus in MT2 cells by entry inhibitors**

Entry inhibitor	Concentration	Continuous^a^		Withdrawal	
		CPE^b^	p24	CPE	p24
DS 003	2 nM	3/3^c^	>600 ng/ml^d^	3/3	>600 ng/ml^d^
	20 nM	1/3	variable	3/3	>600 ng/ml
	200 nM	1/3	variable	3/3	>600 ng/ml
AMD3100	0.18 μM	3/3	>600 ng/ml	3/3	>600 ng/ml
	18 μM	1/3	variable	3/3	>600 ng/ml
	180 μM	1/3	variable	3/3	>600 ng/ml
Maraviroc	1.0 nM	3/3	>600 ng/ml	3/3	>600 ng/ml
	10 nM	3/3	>600 ng/ml	3/3	>600 ng/ml
	100 nM	3/3	>600 ng/ml	3/3	>600 ng/ml

MT2 cells were infected by pseudotyped G367R viruses at 4-fold dilutions and grown in the presence of entry inhibitors. The cultures were then split into 2 wells at day 7 with only one of them being maintained in the presence of inhibitors.

^a^Continuous represents the maintainance of the entry inhibitors in the cultures. Withdrawal represents drug withdrawal after 7 days of infection.

^b^CPE = cytopathic effect.

^c^Numbers of samples in which CPE was observed over 5 weeks after infection.

^d^evaluated during 28 days following infection. p24 values of CPE negative samples were lower than listed.

The CXCR4 inhibitor AMD3100 also delayed reversion but less efficiently (Figure 4b). A concentration of 180 μM that was 10,000 times above the IC_50_ of 7.8 nM (as determined in an assay of cell-free infection with WT virus) was needed to delay G367R reversion (Figure 4b). Again, the virus developed resistance quickly (5 weeks after infection) in one of three experiments (Table 5). As expected, Maraviroc did not have any inhibitory effect. Taking together, it appears that G367R reversion requires the entry of the G367R mutant into cells and that replication must precede reversion. This process can be inhibited by entry inhibitors.

### Endocytosis inhibitors can delay G367R reversion

**Figure 5:**
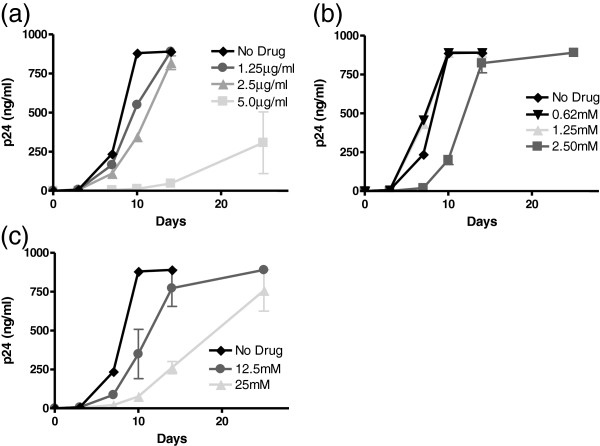
**p24 production in MT2 cells after infection by G367R virus pseudotyped with VSV-G viruses in the presence of endocytosis inhibitors.** MT2 cells were infected for 3 hours at 37°C, then washed with medium, and 10^5^ cells were split into four replicate wells for growth in the presence or absence of chlorpromazine **(a)**, MβCD **(b)** or Genistein **(c)**.

HIV binding to the viral receptor and coreceptor can trigger viral fusion with the plasma membrane; however, the G367R mutant might have lost this ability. One mechanism of HIV entry into cells is dependent on endocytosis [30]. In order to explore whether the spread of the G367R mutant virus might be affected by inhibitors of endocytosis, we employed three such molecules in our experiments, i.e., chlorpromazine which is thought to inhibit clathrin-dependent (CDE) processes as well as two clathrin-independent endocytosis(CIE) inhibitors, i.e., Genistein and methyl-β-cyclodextrin (MβCD). Genistein is a tyrosine-kinase inhibitor and causes local disruption of the actin network at the site of endocytosis while the mechanism of MβCD is to inhibit the endocytosis process that is dependent on the integrity of lipid rafts.All three inhibitors affected cell viability. Only a two-fold higher concentration of chlorpromazine and Genistein resulted in cell death in MT2 than in MT4 cells (data not shown). Only one or two subtoxic concentrations of chlorpromazine and MβCD as tested in 2-fold serial dilutions permitted cell growth yet inhibited G367R virus replication (Figure 5). 5 μg/ml of chlorpromazine slowed increases in p24 values and reversion occurred in only in 2 of 4 experiments after 25 days of infection. Lower concentrations of drug did not inhibit viral reversion. A concentration of 2.5 μM MβCD slowed cell viral growth by about 2-fold and delayed viral replication only slightly. Reversion appeared in all 4 experiments after 25 days of infection (Figure 5b) and lower concentrations of drug had no effect on viral reversion. In contrast, subtoxic concentrations of Genistein inhibited cell growth between 6-40 mM and cells became larger and round, indicating that the drug disrupted the structure of the cell skeleton. However, viral replication was not depressed as much as cell growth because p24 values increased even though the cells grew slowly. Reversion appeared in almost all experiments after 25 days (Figure 5c). These results indicate that the spread of the G367R mutant requires the process of endocytosis and is related to CDE but not to CIE, similar to WT HIV, because this process was not inhibited by MβCD and was not affected as much as Genistein-mediated inhibition of cellular replication.

### Interleukin 2, PMA and ionomycin can block G367R reversion differentially in varying T cell lines

**Figure 6:**
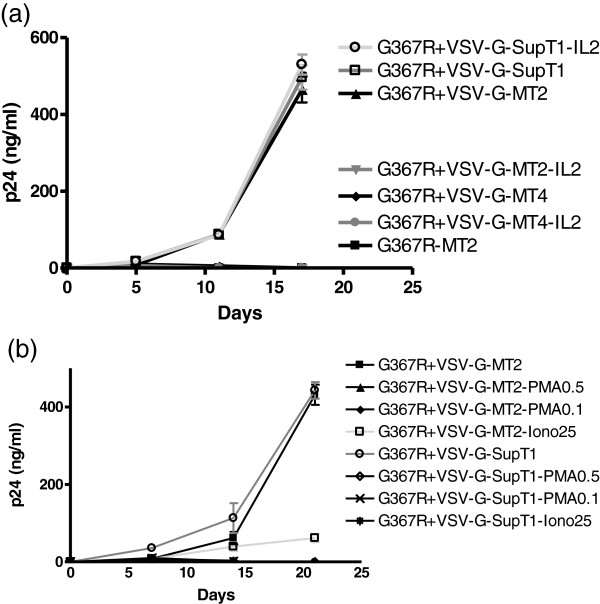
**p24 production in MT2, MT4 and SupT1 cells after infection by G367R virus that had been pseudotyped with VSV-G viruses in the presence of IL2, PMA or ionomycin.** MT2 cells were infected for 3 hours at 37°C, then washed with medium, and 10^5^ cells were split into wells in triplicate for growth in the presence or absence of 20 IU/ml of IL2 **(a)**, 0.1, 0.5 ng/ml of PMA or 25 ng/ml of ionomycin **(b)**.

Based on the above results, G367R reversion may be determined by undefined cellular mechanisms. Contrary to expectations, we found that IL2 was able to inhibit the reversion of G367R virus in MT2 cells at the same concentration used to maintain T lymphocyte growth (Figure 6a) without affecting cell viability, while it only slightly inhibited cell-free wt HIV by about 2 fold (data not shown). In contrast, IL2 had no influence on the reversion of G367R in SupT1 cells, based on both p24 measurements and CPE (Figure 6a). Again, there was no growth or reversion of G367R in MT4 cells, suggesting that a different mechanism of reversion of G367R may be involved in different cells.However, PMA and ionomycin inhibited the reversion of G367R virus in both MT2 and SupT1 cells (Figure 6b). A low concentration of PMA of 0.1 ng/ml or ionomycin at 25 ng/ml both inhibited the G367R reversion. These results suggest that the reversion of G367R that is dependent on transmission between cells may be protein kinase C (PKC) related.

## Discussion

HIV uses two receptors for entry unlike other viruses that employ only a single receptor. This double receptor entry process may have evolutionary advantage and HIV infection that occurs via a single receptor seems less efficient [31]. Differences in ability to infect resting T cells also exist between HIV-env dependent and independent (i.e., pseudotyped) virus entry [16, 24]. In contrast to other HIV env mutants that can enter cells via CD4 independent mechanisms [11, 31, 32], we have discovered an env mutant that is defective in regard to cell-free infection but that can enter some target cells when cell-associated. Indeed, the Env G367R virus seems to be able to cause infection only as cell-associated virus.

This finding may shed light on the fact that HIV can spread between cells in the presence of neutralizing antibodies that might completely block the spread of cell-free virus [33]. Although, CD4bs specific antibodies may be ubiquitously elicited during natural infection [25, 34–36], they did not retain neutralization potency during cell-cell viral transmission. HIV can escape antibody responses rapidly, perhaps due to envelope mutagenesis [37–41]. The G367R mutant is suggestive of conformational changes in the env protein but the process of escape is not fully understood. Previous studies showed that the CD4bs mutation D368R within env lost affinity for neutralizing antibodies and that the mutated viruses were also defective as tested in cell-free infections [25–27].

Similar to what has been observed with the G367R mutant, the blocking activity of CD4bs specific antibodies largely compromises free virus but blocking activity is much less in cell-cell transmission, thus allowing virus entry, replication and spread to occur. Partial or incomplete inhibition of infection likely fosters the emergence of mutants and escape as well as viral replication, even though partial selection pressure is still maintained. In vitro studies of the evolution of drug resistance have reached similar conclusions, i.e., resistance may be more common following cell-cell transmission. In support of this, our data here and the results of others [33, 42, 43] have shown that entry inhibitors can inhibit HIV cell-cell spread but only at much higher concentrations than are effective at blocking cell-free transmission.

Differences in sensitivity of G367R virus to entry inhibitors varied by 10–10,000-fold between cell-to-cell transmission and cell-free transmission. G367R, like D368R, changes the local structure around the CD4bs of env, rendering the virus in cell-free form unable to bind CD4. However, the fact that the mutant can still spread in some cell types indicates that mutated env can retain partial functionality under certain conditions, similar to what can happen in the presence of CD4bs specific antibodies or if cellular mechanisms can compensate for a viral defect. Our cell-type dependent results are consistent with recent results that cell line-based differences can occur during cell-to-cell HIV-1 transmission [44].

However, free virus transmission must be an important component of viral spread in infected individuals. Our results demonstrate that selection pressure on free virus transmission is pronounced to the point that 367R reverts to 367G quickly. Obviously, cell-to-cell transmitted viruses must be dependent on free virus transmission to reach anatomically distant sites. The fitness of 367G (i.e. WT) virus is much higher than that of 367R in cell-free as well as cell-to-cell transmission. Therefore, reversion to 367G is the result of natural selection. This may explain why the G367R or D368R mutations have never been observed as cell-free forms in infected individuals or in tissue culture.

Cell-type differences may also explain why G367R can spread between some cell types, e.g., MT2 and SupT1, but not others. Our finding that a G367R reversion is cell type dependent supports the idea that viral involvement in cell-associated or cell-free transmission depends both on the virus and target cell. Interestingly, MT2 and MT4 cells are both highly sensitive to WT HIV in cell-free infection but behave quite differently in regard to susceptibility to the env mutant G367R in cell-associated entry. The fact that the gp120 binding inhibitor DS003 and the CXCR4 inhibitor AMD3100 can inhibit G367R spread and that the virus can revert in TZM-bl cells but not in 293 T cells indicates that cell-associated transmission of G367R is a receptor-related event. Our findings represent an example of a situation whereby some env mutants such as G367R may be defective in regard to receptor binding but that this can be overcome by cellular mechanisms that compensate and promote cell-associated defective virus to slowly replicate, such that the latter can revert. It is important to note that our study was focused on the G367R mutation in Env and relevant reversion at this site. Of course, it will be important to analyze for other mutations and/or reversions both in Env and in other regions of the viral genome. Such work is complex and is in progress.

The cellular mechanisms underlying the rescue of G367R are complex. MT4 cells are highly sensitive to cell-free WT HIV but do not support cell-associated G367R reversion. Although IL2 can stimulate T cell growth and promote T cell line replication, an unexpected effect of IL2 in regard to G367R reversion was a differential result in varying cell types. However, only IL-2 slightly affected the replication of WT HIV as cell-free virus. Blockage of reversion in MT2 cells is consistent with reports that IL2 can inhibit HIV replication in HTLV-1 transformed T cell lines [45], perhaps due to elevated APOBEC protein incorporation into virions. However, our results with MT4 and SupT1 cells, both of which are Vif permissive, i.e. APOBEC-negative, showed that the G367R virus was able to revert in SupT1 but not in MT4 cells. This suggests that an APOBEC-independent mechanism may be involved and that different mechanisms of G367R reversion may be presenting MT2 vs SupT1 cells (Figure 6a). Protein kinase C (PKC) may be involved in the reversion of G367R because the PKC modulator PMA and the Ca^++^ ionophore ionomycin both inhibited G367R reversion in both MT2 and SupT1 cells (Figure 6b). Chronic PMA treatment of T cell lines may down-regulate PKC [46]. However, PKC is involved in a variety of cellular events and further studies will need to be carried out to clarify the mechanisms involved. We speculate that the HIV envelope may be able to trigger different PKC-mediated events in different cell types, leading to the differential results obtained here, but this needs to be confirmed.

Our data raise the importance of HIV cell-associated forms in the context of viral defectiveness, even in the absence of recombination. As yet undiscovered cellular mechanisms may be involved and HIV may be more fit than studies with cell-free HIV might suggest. It is known that it is easier to isolate clinical viruses via coculture than to recover virus from plasma. Although there are multiple reasons for this, the existence of defective mutants is doubtless one of them.

HIV-infected cells in semen and cervico vaginal secretions —especially infected macrophages and CD4^+^ T cells— play an important role in the sexual transmission of HIV. However, these cells have been largely overlooked in studies of the mechanisms of HIV transmission as well as in the design and testing of HIV vaccine and microbicide candidates, that have mostly evaluated cell-free virus stocks [47, 48]. This explains why some candidates may not protect against cell-associated viral transmission and the failure of several vaccine and microbicide clinical trials.

## Abbreviations

CD4: Cluster of differentiation 4
CD4bs: CD4 binding site
CDE: Clathrin-dependent
CIE: Clathrin-independent endocytosis
CBMCs: Cord blood mononuclear cells
CPE: Cytopathic effects
CCR5: C-C chemokine receptor type 5
CXCR4: C-X-C chemokine receptor type 4
HTLV-1: Human T-Lymphotropic Virus Type 1
MβCD: Methyl-β-cyclodextrin
PKC: Protein kinase C
PMA: Phorbol 12-myristate 13-acetate
VSV-G: G glycoprotein of vesicular stomatitis virus
WT: Wild type.

## References

[1] Sourisseau M, Sol-Foulon N, Porrot F, Blanchet F, Schwartz O (2007). Inefficient human immunodeficiency virus replication in mobile lymphocytes. J Virol.

[2] Chen P, Hubner W, Spinelli MA, Chen BK (2007). Predominant mode of human immunodeficiency virus transfer between T cells is mediated by sustained Env-dependent neutralization-resistant virological synapses. J Virol.

[3] Carr JM, Hocking H, Li P, Burrell CJ (1999). Rapid and efficient cell-to-cell transmission of human immunodeficiency virus infection from monocyte-derived macrophages to peripheral blood lymphocytes. Virology.

[4] Sato H, Orenstein J, Dimitrov D, Martin M (1992). Cell-to-cell spread of HIV-1 occurs within minutes and may not involve the participation of virus particles. Virology.

[5] Sattentau QJ, Weiss RA (1988). The CD4 antigen: physiological ligand and HIV receptor. Cell.

[6] Feng Y, Broder CC, Kennedy PE, Berger EA (1996). HIV-1 entry cofactor: functional cDNA cloning of a seven-transmembrane, G protein-coupled receptor. Science.

[7] Alkhatib G, Combadiere C, Broder CC, Feng Y, Kennedy PE, Murphy PM, Berger EA (1996). CC CKR5: a RANTES, MIP-1alpha, MIP-1beta receptor as a fusion cofactor for macrophage-tropic HIV-1. Science.

[8] Klasse PJ (2012). The molecular basis of HIV entry. Cell Microbiol.

[9] Geijtenbeek TB, Torensma R, van Vliet SJ, van Duijnhoven GC, Adema GJ, van Kooyk Y, Figdor CG (2000). Identification of DC-SIGN, a novel dendritic cell-specific ICAM-3 receptor that supports primary immune responses. Cell.

[10] Kaslow RA, Carrington M, Apple R, Park L, Muñoz A, Saah AJ, Goedert JJ, Winkler C, O'Brien SJ, Rinaldo C, Detels R, Blattner W, Phair J, Erlich H, Mann DL (1996). Influence of combinations of human major histocompatibility complex genes on the course of HIV-1 infection. Nat Med.

[11] Hoffman TL, LaBranche CC, Zhang W, Canziani G, Robinson J, Chaiken I, Hoxie JA, Doms RW (1999). Stable exposure of the coreceptor-binding site in a CD4-independent HIV-1 envelope protein. Proc Natl Acad Sci U S A.

[12] Vidricaire G, Gauthier S, Tremblay MJ (2007). HIV-1 infection of trophoblasts is independent of gp120/CD4 Interactions but relies on heparan sulfate proteoglycans. J Infect Dis.

[13] Tuyama AC, Hong F, Saiman Y, Wang C, Ozkok D, Mosoian A, Chen P, Chen BK, Klotman ME, Bansal MB (2010). Human immunodeficiency virus (HIV)-1 infects human hepatic stellate cells and promotes collagen I and monocyte chemoattractant protein-1 expression: implications for the pathogenesis of HIV/hepatitis C virus-induced liver fibrosis. Hepatology.

[14] Domingo E, Holland JJ (1997). RNA virus mutations and fitness for survival. Annu Rev Microbiol.

[15] Meyerhans A, Cheynier R, Albert J, Seth M, Kwok S, Sninsky J, Morfeldt-Manson L, Asjo B, Wain-Hobson S (1989). Temporal fluctuations in HIV quasispecies in vivo are not reflected by sequential HIV isolations. Cell.

[16] Quan Y, Brenner BG, Dascal A, Wainberg MA (2008). Highly diversified multiply drug-resistant HIV-1 quasispecies in PBMCs: a case report. Retrovirology.

[17] Quan Y, Liang C, Brenner BG, Wainberg MA (2009). Multidrug-resistant variants of HIV type 1 (HIV-1) can exist in cells as defective quasispecies and be rescued by superinfection with other defective HIV-1 variants. J Infect Dis.

[18] Li Y, Kappes JC, Conway JA, Price RW, Shaw GM, Hahn BH (1991). Molecular characterization of human immunodeficiency virus type 1 cloned directly from uncultured human brain tissue: identification of replication-competent and -defective viral genomes. J Virol.

[19] Dang Q, Chen J, Unutmaz D, Coffin JM, Pathak VK, Powell D, KewalRamani VN, Maldarelli F, Hu WS (2004). Nonrandom HIV-1 infection and double infection via direct and cell-mediated pathways. Proc Natl Acad Sci U S A.

[20] Del Portillo A, Tripodi J, Najfeld V, Wodarz D, Levy DN, Chen BK (2011). Multiploid inheritance of HIV-1 during cell-to-cell infection. J Virol.

[21] Onafuwa-Nuga A, Telesnitsky A (2009). The remarkable frequency of human immunodeficiency virus type 1 genetic recombination. Microbiol Mol Biol Rev.

[22] Sigal A, Kim JT, Balazs AB, Dekel E, Mayo A, Milo R, Baltimore D (2011). Cell-to-cell spread of HIV permits ongoing replication despite antiretroviral therapy. Nature.

[23] Mostowy R, Kouyos RD, Fouchet D, Bonhoeffer S (2011). The role of recombination for the coevolutionary dynamics of HIV and the immune response. PLoS One.

[24] Yu D, Wang W, Yoder A, Spear M, Wu Y (2009). The HIV envelope but not VSV glycoprotein is capable of mediating HIV latent infection of resting CD4 T cells. PLoS Pathog.

[25] Li Y, Migueles SA, Welcher B, Svehla K, Phogat A, Louder MK, Wu X, Shaw GM, Connors M, Wyatt RT, Mascola JR (2007). Broad HIV-1 neutralization mediated by CD4-binding site antibodies. Nat Med.

[26] Kwong PD, Wyatt R, Robinson J, Sweet RW, Sodroski J, Hendrickson WA (1998). Structure of an HIV gp120 envelope glycoprotein in complex with the CD4 receptor and a neutralizing human antibody. Nature.

[27] Thali M, Olshevsky U, Furman C, Gabuzda D, Li J, Sodroski J (1991). Effects of changes in gp120-CD4 binding affinity on human immunodeficiency virus type 1 envelope glycoprotein function and soluble CD4 sensitivity. J Virol.

[28] Mochizuki N, Otsuka N, Matsuo K, Shiino T, Kojima A, Kurata T, Sakai K, Yamamoto N, Isomura S, Dhole TN, Takebe Y, Matsuda M, Tatsumi M (1999). An infectious DNA clone of HIV type 1 subtype C. AIDS Res Hum Retroviruses.

[29] Quan Y, Xu H, Wainberg MA (2014). Defective HIV-1 quasispecies in the form of multiply drug-resistant proviral DNA within cells can be rescued by superinfection with different subtype variants of HIV-1 and by HIV-2 and SIV. J Antimicrob Chemother.

[30] Miyauchi K, Kim Y, Latinovic O, Morozov V, Melikyan GB (2009). HIV enters cells via endocytosis and dynamin-dependent fusion with endosomes. Cell.

[31] Dumonceaux J, Nisole S, Chanel C, Quivet L, Amara A, Baleux F, Briand P, Hazan U (1998). Spontaneous mutations in the env gene of the human immunodeficiency virus type 1 NDK isolate are associated with a CD4-independent entry phenotype. J Virol.

[32] Chenine AL, Pion M, Matouskova E, Gondois-Rey F, Vigne R, Hirsch I (2002). Adaptation of a CXCR4-using human immunodeficiency type 1 NDK virus in intestinal cells is associated with CD4-independent replication. Virology.

[33] Abela IA, Berlinger L, Schanz M, Reynell L, Gunthard HF, Rusert P, Trkola A (2012). Cell-cell transmission enables HIV-1 to evade inhibition by potent CD4bs directed antibodies. PLoS Pathog.

[34] Li Y, Svehla K, Louder MK, Wycuff D, Phogat S, Tang M, Migueles SA, Wu X, Phogat A, Shaw GM, Connors M, Hoxie J, Mascola JR, Wyatt R (2009). Analysis of neutralization specificities in polyclonal sera derived from human immunodeficiency virus type 1-infected individuals. J Virol.

[35] Scheid JF, Mouquet H, Ueberheide B, Diskin R, Klein F, Oliveira TY, Pietzsch J, Fenyo D, Abadir A, Velinzon K, Hurley A, Myung S, Boulad F, Poignard P, Burton DR, Pereyra F, Ho DD, Walker BD, Seaman MS, Bjorkman PJ, Chait BT, Nussenzweig MC (2011). Sequence and structural convergence of broad and potent HIV antibodies that mimic CD4 binding. Science.

[36] Dhillon AK, Donners H, Pantophlet R, Johnson WE, Decker JM, Shaw GM, Lee FH, Richman DD, Doms RW, Vanham G, Burton DR (2007). Dissecting the neutralizing antibody specificities of broadly neutralizing sera from human immunodeficiency virus type 1-infected donors. J Virol.

[37] Chen L, Kwon YD, Zhou T, Wu X, O'Dell S, Cavacini L, Hessell AJ, Pancera M, Tang M, Xu L, Yang ZY, Zhang MY, Arthos J, Burton DR, Dimitrov DS, Nabel GJ, Posner MR, Sodroski J, Wyatt R, Mascola JR, Kwong PD (2009). Structural basis of immune evasion at the site of CD4 attachment on HIV-1 gp120. Science.

[38] Richman DD, Wrin T, Little SJ, Petropoulos CJ (2003). Rapid evolution of the neutralizing antibody response to HIV type 1 infection. Proc Natl Acad Sci U S A.

[39] Wei X, Decker JM, Wang S, Hui H, Kappes JC, Wu X, Salazar-Gonzalez JF, Salazar MG, Kilby JM, Saag MS, Komarova NL, Nowak MA, Hahn BH, Kwong PD, Shaw GM (2003). Antibody neutralization and escape by HIV-1. Nature.

[40] Frost SD, Wrin T, Smith DM, Kosakovsky Pond SL, Liu Y, Paxinos E, Chappey C, Galovich J, Beauchaine J, Petropoulos CJ, Little SJ, Richman DD (2005). Neutralizing antibody responses drive the evolution of human immunodeficiency virus type 1 envelope during recent HIV infection. Proc Natl Acad Sci U S A.

[41] Trkola A, Kuster H, Rusert P, Joos B, Fischer M, Leemann C, Manrique A, Huber M, Rehr M, Oxenius A, Weber R, Stiegler G, Vcelar B, Katinger H, Aceto L, Günthard HF (2005). Delay of HIV-1 rebound after cessation of antiretroviral therapy through passive transfer of human neutralizing antibodies. Nat Med.

[42] Permanyer M, Ballana E, Badia R, Pauls E, Clotet B, Este JA (2012). Trans-infection but not infection from within endosomal compartments after cell-to-cell HIV-1 transfer to CD4+ T cells. J Biol Chem.

[43] Ayouba A, Cannou C, Nugeyre MT, Barre-Sinoussi F, Menu E (2008). Distinct efficacy of HIV-1 entry inhibitors to prevent cell-to-cell transfer of R5 and X4 viruses across a human placental trophoblast barrier in a reconstitution model in vitro. Retrovirology.

[44] Zhong P, Agosto LM, Ilinskaya A, Dorjbal B, Truong R, Derse D, Uchil PD, Heidecker G, Mothes W (2013). Cell-to-cell transmission can overcome multiple donor and target cell barriers imposed on cell-free HIV. PLoS One.

[45] Oguariri RM, Dai L, Adelsberger JW, Rupert A, Stevens R, Yang J, Huang D, Lempicki RA, Zhou M, Baseler MW, Lane HC, Imamichi T (2013). Interleukin-2 inhibits HIV-1 replication in some human T cell lymphotrophic virus-1-infected cell lines via the induction and incorporation of APOBEC3G into the virion. J Biol Chem.

[46] Ahnadi CE, Giguere P, Gravel S, Gagne D, Goulet AC, Fulop T, Payet MD, Dupuis G (2000). Chronic PMA treatment of Jurkat T lymphocytes results in decreased protein tyrosine phosphorylation and inhibition of CD3- but not Ti-dependent antibody-triggered Ca2+ signaling. J Leukoc Biol.

[47] Kolodkin-Gal D, Hulot SL, Korioth-Schmitz B, Gombos RB, Zheng Y, Owuor J, Lifton MA, Ayeni C, Najarian RM, Yeh WW, Asmal M, Zamir G, Letvin NL (2013). Efficiency of cell-free and cell-associated virus in mucosal transmission of human immunodeficiency virus type 1 and simian immunodeficiency virus. J Virol.

[48] Bubnoff AV: **Is HIV Hitching a Ride Inside Cells?** IAVI Report 2011 VOL. 15, NO. 1 21449504

